# Palliative Radiotherapy at the Crossroads of Supportive Oncology: Addressing Global Gaps, Guideline Deficits, and the Expanding Need for Symptom-Directed Cancer Care

**DOI:** 10.3390/cancers18040564

**Published:** 2026-02-09

**Authors:** Beth Chasty, Richard Berman, Ashique Ahamed, Edward Chow, Agata Rembielak, Eva Oldenburger

**Affiliations:** 1 The Christie NHS Foundation Trust, Manchester M20 4BX, UKashique.ahamed@nhs.net (A.A.); agata.rembielak@nhs.net (A.R.); 2 Division of Cancer Sciences, School of Medical Sciences, Faculty of Biology, Medicine and Health, The University of Manchester, Manchester M13 9PL, UK; 3 Department of Radiation Oncology, Sunnybrook Health Sciences Centre, University of Toronto, Toronto, ON M4N 3M5, Canada; 4 Department of Radiation Oncology, University Hospitals Leuven, 3000 Leuven, Belgium; eva.oldenburger@uzleuven.be; 5 Department of Palliative Care, University Hospitals Leuven, 3000 Leuven, Belgium

**Keywords:** palliative radiotherapy, supportive oncology, toxicity, late effects, survivorship, senior adult oncology

## Abstract

Radiotherapy has been a central component of cancer therapy for over a century, with the field rapidly evolving over time, resulting in improved outcomes. These advances have contributed to a changing demographic, with growing numbers of people living longer with, and surviving the disease. This has been accompanied by an increasing burden of chronic physical and psychological side effects, including radiotherapy-related toxicity. These long-term consequences have a substantial impact on patients, their carers, and healthcare systems. Significant global inequities persist, particularly in low- and middle-income countries (LMICs), where access to radiotherapy and comprehensive supportive and palliative care remains limited. Supportive oncology has emerged as a new field focusing on the management of acute, chronic, and emergent problems in people living with and beyond cancer. Despite its benefits, palliative radiotherapy continues to be underused, especially in specific patient groups including paediatrics, older age adults, and those with a short prognosis. Often treatment decisions in these patient groups are challenging and the integration of supportive oncology could help overcome this. Furthermore, radiotherapy toxicity and its management has been under-researched despite huge advancements in cancer treatments leading to a lack of guidelines and varied practice globally. Supportive oncology offers a framework to address these challenges through earlier integration into treatment pathways, multidisciplinary collaboration, and a stronger focus on symptom control, survivorship, and equity. Embedding supportive oncology within radiotherapy services is essential to delivering high-quality, patient-centred cancer care worldwide.

## Simple Summary

Palliative radiotherapy has a wide range of applications in the treatment of advanced cancers. It is a key part of the management of several oncology emergencies including metastatic spinal cord compression and superior vena cava obstruction. It can provide symptom control but also halt progression of disease. Despite its benefits, it is underutilised globally. Advances in radiotherapy techniques have improved long-term survival. There are therefore many people living with and beyond cancer. However, alongside this, there is a rise in the number of people living with significant toxicity from treatment including those treated with radical or palliative radiotherapy. This paper highlights the role of supportive oncology in tackling both palliative radiotherapy underutilisation and radiotherapy toxicity by promoting its integration into radiotherapy services. This paper identifies the need for supportive oncology-focused research, education, and guidelines on radiotherapy toxicity management and outlines areas for future development.

## 1. Introduction

Supportive oncology, as defined by the Multinational Association of Supportive Care in Cancer (MASCC), addresses the prevention and management of adverse effects of cancer and its treatment. This includes management of physical and psychological symptoms and side effects across the continuum of the cancer journey from diagnosis through treatment to post-treatment care. Supportive care aims to improve the quality of rehabilitation, secondary cancer prevention, survivorship, and end-of-life care [1].

As cancer therapies evolve and survival improves, supportive oncology has emerged as an essential and rapidly developing specialty. Increasing numbers of patients now live with treatable but incurable disease or as long-term survivors, often with complex unmet needs [2]. Optimal supportive oncology can assist with accurate diagnosis and management and consequently improves outcomes. It requires input from a broad range of medical (and non-medical specialties) outside of oncology, working in an integrated way [3]. The supportive oncology multi-disciplinary team (MDT) includes but is not limited to medical oncology, clinical/radiation oncology, palliative medicine, senior adult oncology, acute oncology, psycho-oncology, cardio-oncology, endo-oncology, rehabilitation oncology, integrative medicine, pharmacy, dietetics, physio and occupational therapy.

The global cancer burden continues to grow. The WHO estimated 22 million new cases of cancer worldwide in 2022; projected to rise by 77% to 35 million new cases by 2050. This highlights the urgent capacity demands for oncology services [4]. This need has also been acknowledged by several professional organisations in the UK, through position statements by the Royal College of Physicians (RCP) and the Royal College of Radiologists (RCR) highlighting the need for the integration and funding of acute and supportive oncology services [5,6]. Health economic assessments further support the argument for allocating or reallocating resources towards supportive oncology service development [7]. Furthermore, supportive oncology is not yet embedded within formal oncology training, and this is an area for future development.

As cancer survival improves, increasing number of patients are living with long-term cancer and treatment-related consequences, including those receiving surgery, radiotherapy, and systemic anticancer therapy (SACT). In the UK alone, approximately 25% of treated patients experience persistent side effects that impair function and quality of life [8]. Although the importance of comprehensive supportive and survivorship care is widely acknowledged, most notably in the ASCO–ESMO quality cancer care consensus, its delivery remains inconsistent [9]. This gap between recognised need and service provision underscores the importance of embedding supportive oncology within routine cancer care pathways.

Radiotherapy represents a major component of modern cancer care with more than 50% of cancer patients receiving radiotherapy as part of their treatment [10,11]. It is a treatment modality relevant to all stages of the disease: curative, adjuvant, neoadjuvant, definitive, and metastatic, and can be delivered either as stand-alone radiotherapy or in combination with systemic therapy [12,13,14,15]. Palliative radiotherapy plays a pivotal role in symptom management, offering effective relief for pain, bleeding, obstruction, and other cancer-related complications. Despite this, palliative radiotherapy continues to be underused globally, especially in specific patient groups including paediatrics, older age adults, and those with a short prognosis [16].

Given its potential for widespread use and impact on both survival and symptom burden, radiotherapy offers a critical opportunity for the integration of supportive oncology principles into everyday clinical practice. Consequently, radiation oncologists, as well as clinical oncologists with expertise in radiation therapy, are well positioned to be frontrunners in advancing and actively contributing to supportive oncology.

Despite technique advances and its use across multiple tumour sites, there is still a lack of research and clinical guidelines in the management of side effects, particular late effects [17]. This continues to have a significant impact on patients long after radiotherapy and contributes to long-term morbidity and healthcare utilisation. This represents a key area in which supportive oncology can add substantial value, through systematic assessment, early intervention, and coordinated long-term follow-up for patients receiving radiotherapy.

## 2. Development and Current Role of Palliative Radiotherapy (PRT)

The late 19th century saw the discovery of x-rays and soon following this, the first use of radiotherapy in the palliation of cancer symptoms [18,19,20]. Advances throughout the 20th century have led to PRT becoming a well-established and effective way of treating the symptoms associated with advanced and metastatic cancer [18]. It is generally well-tolerated, non-invasive, and enables symptoms to be quickly controlled, leading to an improvement in quality of life, and is therefore considered an appropriate treatment for those with a poor performance status or short prognosis [21].

## 3. Definition of Palliative Radiotherapy

PRT has been used to describe the use of x-rays to improve symptoms caused by advanced and metastatic cancer alongside its role in providing local tumour control [22]. Advances in radiotherapy techniques have enabled better dose conformation leading to improved outcomes and toxicity profiles. Those with previously incurable disease are now seeing long-term survival or even cure with the use of stereotactic ablative body radiotherapy (SABR) in oligometastatic cancers [14,15]. The potential of PRT to offer improved disease-free survival has blurred the lines between palliative and curative treatment [18,23]. Furthermore, there is a misconception of the word ‘palliative’, with many patients and their families associating it with the discontinuation of treatment and end-of-life care [24]. This raises the question as to whether an alternative name would be better to describe this treatment. One consideration is ‘supportive’ radiotherapy, which would remove the association with end-of-life care, and bring it into line with modern rebranding, reflecting the changed demographic. Kopecky and Campbell have suggested re-naming palliative intent systemic treatments as ‘non-curative’ whilst Neugut and Prigerson propose the phrase ‘life-extending’ chemotherapy with the aim of communicating a clear message to patients regarding the intent of treatment [25,26]. Oncologists’ perception of palliative care is also important as it impacts referral practices [27]. A change from the name palliative care to supportive care in one cancer centre increased inpatient referrals and saw outpatient referrals being made earlier [28]. This subtle but important change may have led to improved patient outcomes through earlier supportive care involvement.

## 4. Fractionation

Supported by clinical evidence, international guidelines for PRT consistently recommend the use of short hypofractionated regimes with common schedules being an 8 Gy single fraction, 20 Gy in five fractions, and 30 Gy in ten fractions [29,30,31,32]. Hypofractionation reduces treatment burden, healthcare costs, and hospital visits while allowing individualisation based on tumour biology, prognosis, and patient preference; all principles aligned with supportive oncology.

In bone metastases, randomised trials and meta-analyses have demonstrated equivalent pain relief and toxicity profiles across fractionation schedules, although re-irradiation rates are higher following single-fraction treatment [33,34,35,36]. This trade-off must be considered within the context of prognosis and performance status, as the reduced treatment burden of a single fraction may be preferable for many patients. Despite strong evidence and guideline endorsement, single-fraction PRT remains underutilised internationally [33,37,38,39]. International guidance including that by the RCR in the UK, ESTRO ARCOP, and ASTRO recommend single fraction PRT bone metastases, and it is felt that this momentum should lead to changes in global practice [30,31,36].

## 5. Clinical Applications Across Disease Sites

PRT has broad applications in advanced cancer, providing rapid relief from pain, dyspnoea, neurological deficits, bleeding, obstruction, and ulcerating or fungating tumours [16,40].

Bone metastases are a common presentation in metastatic disease and are the third most common site of metastasis in solid organ cancers [41]. They can lead to complications such as pain, hypercalcaemia, and fractures resulting in disability. Radiotherapy offers a rapid, efficient means of treatment for pain, with 60–80% of patient seeing pain control within 3–4 weeks [41,42]. One study has also highlighted the significant quality of life improvement seen with the use of radiotherapy in painful bone metastases within 10 days, supporting the use of PRT for symptom control even for patients with a short prognosis [43].

Post-operative radiotherapy following decompression for metastatic spinal cord compression improves functional outcomes and reduces the risk of further intervention [30,44]. Similarly, in non-spinal complicated bone metastases, post-operative radiotherapy enhances local control and reduces re-operation rates by treating any residual disease and stabilising the surgical hardware [34,45]. Commonly used fractionations are 8 Gy single fraction, 20 Gy in five fractions or 30 Gy in 10 fractions with the advice that this takes place within 5 weeks of surgery whilst allowing for wound healing [34]. In combination, surgery and PRT improve both outcomes and pain, consequently improving a patients’ quality of life. It can be also used as the sole treatment of pathological fractures not amenable to surgery such as rib, pelvis, vertebrae, and shoulder girdle [29].

Radiotherapy can also be used prophylactically to reduce risk of fracture in areas felt to be at risk [29]. This aims to reduce the pain and morbidity associated with a fracture with a recent study providing evidence to support this along with evidence of improved overall survival [46].

Vertebroplasty, kyphoplasty, bisphosphonates, and radionucleotides are other therapies used in the treatment of bone metastases, but their use has been recommended alongside radiotherapy [47]. This highlights that despite advances in treatments, PRT continues to be relevant and essential.

PRT is a key part of the management of several oncology emergencies including metastatic spinal cord compression, superior vena cava obstruction, bronchial obstruction, tumour haemorrhage, and in the setting of increased intracranial pressure secondary to brain metastases [40,48]. MDT involvement is essential to ensure a prompt decision is made to determine the optimal treatment for each patient with surgery, interventional radiology, endoscopic interventions and chemotherapy being alternative strategies [49]. Untreated oncological emergencies can lead to significant morbidity and risk to life. PRT is a less intrusive treatment suitable for those with a poorer performance status and prognosis aiming to improve quality of life in these patient groups.

PRT can also be used prophylactically to halt disease progression and prevent the onset of symptoms associated with disease. Its use has been seen in range of disease sites including bone and lung [46,50] and has been demonstrated to reduce hospital visits [46]. This not only has a significant benefit to patients but also for healthcare providers. Its use is also seen in the primary treatment of malignancies for those with significant frailty unable to undergo surgery, or intensive systemic therapy or radiotherapy regimes [51,52,53].

Brain metastases affect 20–40% of cancer patients and are associated with a significant reduction in both survival and quality of life [54,55]. Whole-brain radiotherapy (WBRT) alongside dexamethasone has been a key treatment in the management of brain metastases since the 1970s as it can relieve symptoms and improve intracranial disease control leading to an improvement in survival [56]. It has also been used post-operatively to reduce the risk of recurrence [29]. Survival without treatment is estimated at 1 to 2 months [57] with median overall survival after WBRT ranging between 3 and 6 months depending on patient characteristics [58]. However, WBRT has been associated with significant side effects including an increase in pain, fatigue, drowsiness, reduced appetite, and neurocognitive decline, all contributing to a reduction in quality of life [55,59]. Hippocampal sparing and use of memantine are all techniques used to reduce risk of neurocognitive decline [59,60]. A recent phase three randomised trial in patients with non-small-cell lung cancer and brain metastases highlighted there was no significant difference in quality of life and survival outcomes in patients treated with WBRT versus best supportive care [61]. Treatment decisions in this patient cohort are challenging and integration of supportive oncology may help support patients through this intense treatment whilst also identifying cases where best supportive care may be more appropriate.

Recent advances have seen stereotactic radiosurgery at the forefront of treatment for brain metastasis which reduces the dose to healthy brain tissue. It can be used in cases of up to 15 brain metastases with similar outcomes compared to WBRT, with reduced risk of neurocognitive decline and shorter treatment time [62,63]. Recent studies are looking at its use in cases of higher metastatic burden and further development in this field could lead to a decline in the use of WBRT [64].

## 6. Radiotherapy and Its Toxicity

### 6.1. Acute Toxicity

Despite significant technological advances, radiotherapy remains associated with both acute and late toxicities, resulting in substantial physical and psychosocial burdens for patients and healthcare systems [65,66]. Notably, there is a paucity of robust data quantifying the proportion of patients affected by radiotherapy-related side effects across tumour sites, reflecting a broader lack of research and systematic surveillance in this area.

Acute toxicities arise from damage to rapidly proliferating normal tissues, such as epithelial cells, and typically develop one to two weeks after the initiation of radiotherapy, often peaking several weeks following treatment completion [16,67]. These effects are frequently reversible and commonly include fatigue, dermatitis, mucositis, oedema, oesophagitis, and diarrhoea. Subacute toxicities, such as pneumonitis, neurological deficits, nephrotoxicity, and vertebral body fractures, may occur weeks to months after treatment.

Although comprehensive data across tumour sites are limited, acute toxicities during radiotherapy are highly prevalent. Disease-specific rates are substantial: up to 84% of cervical cancer patients experience acute toxicity [68], 74–100% of breast radiotherapy patients develop skin reactions [69], and 93% of prostate radiotherapy patients experience genitourinary symptoms [70].

Acute toxicities have important clinical consequences. Poorly controlled symptoms lead to treatment interruptions, with up to 25% of patients missing radiotherapy sessions, potentially reducing tumour control and overall survival [16,71].

Fatigue is among the most common and debilitating acute toxicities, affecting up to 80% of patients during radiotherapy and significantly impairing quality of life [72,73]. Mechanisms underlying radiotherapy-related fatigue remain poorly understood, and its impact is often underestimated, resulting in limited targeted interventions [73]. Effective management strategies for acute radiotherapy-related fatigue include exercise, relaxation therapy, sleep optimisation, nutritional assessment, and group-based psychological interventions [73,74].

One study reported that more than 50% of patients experienced significant physical concerns during radiotherapy—including pain, fatigue, sleep disturbance, appetite changes, and reduced physical function—as well as emotional distress such as anxiety, fear, and worry [75]. Importantly, substantial discordance between clinician-reported and patient-reported toxicity has been consistently demonstrated, suggesting that symptom burden is frequently under-recognised in routine clinical practice [76].

### 6.2. Late Effects

Late effects result from injury to tissues with slower cellular turnover, including the central nervous system, kidneys, heart, and vasculature, causing fibrosis, atrophy, necrosis, and vascular damage. Symptoms including neuropathy, strictures, chronic pain, cardiac injury, gastrointestinal and genitourinary dysfunction and an increased risk of secondary malignancies [77,78]. These toxicities occur more than three months post-treatment and are often irreversible and progressive, with a significant impact on long-term function and quality of life [79,80]. As a result, radiotherapy dose constraints are largely based on the risk of late effects and permanent organ damage [77]. Late effects also include psychological symptoms [80]. Approximately 5–10% of patients experience severe late toxicity following radiotherapy and with increasing global cancer incidence and improved survival, the absolute number of affected individuals continues to rise, constituting a growing public health challenge [81]. Despite this, late effects remain under-recognised, under-researched, and poorly managed, with limited evidence-based guidelines to inform clinical practice [16]. While newer modalities such as SABR and proton beam therapy aim to improve tumour control and reduce toxicity, long-term outcome data remains limited due to their relatively recent adoption [80].

Pelvic radiotherapy represents one of the most common radiotherapy indications and is associated with particularly burdensome late gastrointestinal and genitourinary toxicities, including incontinence and sexual dysfunction [82]. Up to 90% of patients report permanent changes in bowel habit following pelvic radiotherapy, with approximately 50% experiencing a significant reduction in quality of life [83]. The term *pelvic radiation disease* has been introduced to describe the spectrum of transient and long-term toxicities affecting non-cancerous tissues following pelvic radiotherapy, including symptoms involving the bowel, urinary tract, sexual organs, bones, and skin [82]. Surveys of UK clinical oncologists indicate that pelvic radiation disease is widely perceived as a significant clinical problem but remains under-recognised and inadequately managed [82,84]. Consequently, only around 20% of patients with bowel toxicity are referred for gastroenterology assessment, despite the availability of effective interventions [83]. In response to these gaps in care, the Pelvic Radiation Disease Association (PRDA) was established to improve awareness and provide guidance for patients and healthcare professionals [85].

The long-term impact of radiotherapy-related toxicity is reflected in survivorship outcomes. Within the NHS, approximately one-third of cancer survivors report a poor quality of life following treatment [86]. A large German study involving 1348 cancer survivors four years after radiotherapy or systemic anticancer therapy found that only 16.3% were symptom-free, with many reporting inadequate post-treatment support [87]. The landmark Institute of Medicine report: *From Cancer Patient to Cancer Survivor: Lost in Transition* highlighted significant deficiencies in survivorship care and recommended the implementation of personalised survivorship care plans [88]. However, despite these recommendations, adoption of survivorship care plans remains limited in routine practice [89]. More recently, ESMO has emphasised that comprehensive survivorship care should extend beyond the management of physical toxicities to include psychological, social, occupational, and financial well-being, surveillance for recurrence and secondary malignancies, and promotion of overall health [90]. MASCC and ASCO have further recognised that survivorship care should also encompass patients with advanced and metastatic cancer, reflecting improving outcomes in these populations [91]. Nevertheless, substantial geographical inequalities in survivorship care persist globally, compounded by limited training for healthcare professionals and insufficient funding for service provision [92,93,94,95].

The emergence of supportive oncology provides a framework to address these challenges by promoting standardised toxicity assessment, early intervention, multidisciplinary collaboration, and equitable access to survivorship services. Integrating supportive oncology into radiotherapy pathways offers a critical opportunity to mitigate the long-term burden of treatment-related toxicity and improve quality of life for patients living with and beyond cancer.

### 6.3. Toxicity Management in Palliative Radiotherapy

PRT is generally well tolerated particularly when compared with radiotherapy delivered with curative intent and is widely used in metastatic disease [20,96].

As radiotherapy-related toxicity is largely determined by dose to surrounding organs at risk (OARs), many side effects are predictable and potentially preventable [96]. Appropriate patient counselling and anticipatory symptom management are therefore essential components of care.

Prophylactic measures, including analgesia and anti-emetics, are frequently employed to reduce treatment-related toxicity [96,97]. Pain flare following PRT for bone metastases is a well-recognised phenomenon, occurring in approximately 40% of patients [98,99]. It typically develops within five days of treatment initiation and lasts a median of three days, with evidence supporting the use of dexamethasone for prophylaxis [98]. Unfortunately, there is limited randomised evidence to guide management of other symptoms following PRT, resulting in wide variation in the use of supportive interventions such as skin care, analgesia, anti-inflammatory therapy, bowel and airway management, and psychological support [80]. These gaps highlight a clear role for supportive oncology in PRT, with an emphasis on standardised toxicity assessment, proactive symptom management, multidisciplinary coordination, and informed decision-making, particularly in patients with limited prognosis, where treatment appropriateness and patient-centred goals of care must be carefully balanced.

### 6.4. Radiotherapy Toxicity and the Role of Supportive Oncology

The increasing use of patient-reported outcome measures (PROMs) in radiotherapy has improved symptom detection and communication between patients and clinicians, leading to reductions in symptom burden and, in some studies, improvements in survival outcomes [76,100,101,102].

Structured supportive care programmes implemented in radiotherapy departments, particularly in American cancer centres, have demonstrated a 40% reduction in missed appointments [103]. Prehabilitation strategies aimed at optimising patients before radiotherapy by addressing physical fitness, nutrition, and psychological resilience, have also been identified as a promising approach to reducing toxicity and improving treatment adherence, although further research is required [104]. The integration of supportive oncology within radiotherapy services enables early identification of acute toxicities and emotional distress, improves symptom control, enhances treatment adherence, and consequently improves patient-reported outcomes and quality of life. The importance of supportive care during radiotherapy is increasingly recognised internationally. Expert consensus from ESTRO and ESMO highlights the need for structured supportive care pathways for patients receiving concurrent chemoradiotherapy for lung cancer [105]. Despite this growing recognition, there remains a need for further supportive oncology-focused research, including prospective clinical trials, to define optimal strategies for the prevention and management of radiotherapy-related toxicities [106].

## 7. Re-Irradiation

Re-irradiation is taking place more frequently due to the improved survival associated with advances in cancer treatments and developments in radiotherapy delivery leading to improved toxicity profiles. Re-irradiation can take place in the setting of recurrent, metastatic or new disease [107]. The decision to offer re-irradiation is challenging based on balancing the risk of progressive disease versus the risk of severe toxicity [107]. There is a lack of evidence and therefore practices vary. In response to this ESTRO/EORTC released a paper in 2022 defining re-irradiation and outlining the essential parameters which require reporting to enable cross study comparison [108,109]. They also highlighted patient and radiobiological factors to consider and the importance of shared and multi-disciplinary decision-making [108]. Supportive oncology professionals can form part of an MDT to help support clinicians and patients in this setting.

## 8. Novelties/Developments—With Opportunities for Supportive Oncology

### 8.1. SABR and Oligometastatic Disease

SABR is changing the landscape of radiotherapy treatment having shown curative potential in select oligometastatic patients [14,15]. Supportive oncology can contribute to

• Monitoring and managing SABR-specific toxicities.
• Quality of life assessment in prolonged survivorship.
• Decision-making regarding aggressive local therapies in frail patients.

### 8.2. MR-Linac and Adaptive Radiotherapy

MR-guided radiotherapy is a new technology that is expanding globally but its use is not widespread. It allows real-time anatomical tracking, enabling

• Reduced toxicity [110].
• Precise targeting near critical structures [111].
• Adaptive palliative treatments for mobile or sensitive lesions.
• Simulation and treatment can take place in one session reducing waiting times for patients, staff involvement, and improving capacity within departments [112].

### 8.3. Spatially Fractionated Radiotherapy (SFRT)

Spatially fractionated radiotherapy (SFRT), including historical GRID (a 2D or 3D approach using physical or virtual grids to create alternating high- and low-dose regions) and modern Lattice radiotherapy (volumetric planning to place multiple high-dose “vertices” within a bulky tumour in three dimensions), may be considered for select patients with bulky, symptomatic tumours as an alternative to conventional palliative radiotherapy to provide timely symptom relief and more adequate OAR sparing [113,114,115]. High spatial dose modulation in SFRT has been found to activate distinct radiobiological mechanisms which increase normal tissues tolerances [116,117]. This improved therapeutic ratio has led to its use in re-irradiation and in cases where stereotactic body radiotherapy (SBRT) is not technically possible due to size restrictions or proximity to OARs [118,119].

However, the current clinical evidence remains heterogeneous and largely non-randomised, with variability in technique, dose, and patient selection [120,121,122]. Accordingly, within a supportive care framework, evaluation of *benefit* should prioritise symptom relief and PROMs, with transparent reporting of acute toxicity, rather than radiographic response alone. 

### 8.4. Emerging Novel Palliative Indications

Examples include:

• SABR to coeliac plexus for intractable pancreatic pain offers a non-invasive alternative to coeliac plexus block [123].
• The use of proton therapy in the palliative setting offering improved toxicity profiles including in the setting of re-irradiation [124].
• Ultra-hypofractionated regimens for rapid symptom relief [125].
• Integration of radiopharmaceuticals with EBRT [126].

## 9. Underusage of Radiotherapy

Globally, PRT is underutilised [39,127,128]. On average, PRT is delivered to 40% of patients with advanced cancer [129]. However, there is a significant number of patients who would benefit from PRT who are not receiving treatment [127]. There are several factors contributing to this gap including geographical inequality, physician education, and referrer bias [130,131]. The rise of supportive oncology should help to improve links between palliative care, medical, and clinical oncologists improving PRT referral pathways.

The rationale for the promotion of PRT within supportive care is its ability to improve quality of life [96]. The disease-related symptoms of advanced cancer can be quickly and effectively managed with limited hospital visits and minimal treatment side effects [96].

Other significant barriers to PRT include geographic distance and socioeconomic status [127,131]. Radiotherapy takes place in cancer centres often located in cities. Studies have highlighted that patients living further from cancer centres are less likely to receive radiotherapy [132,133]. Travel to radiotherapy centres also incurs significant costs which disadvantages those of lower socioeconomic status. Rapid-access PRT clinics have emerged as an effective solution, providing assessment, planning, and treatment within a single day, improving care coordination and promoting evidence-based hypofractionation [134,135].

Radiotherapy and PRT are a key aspect of comprehensive cancer care but are significantly underused in LMICs. A total of 57% of global cancer cases are in LMICs but despite this, there are still 23 LMICs without any active radiotherapy facilities [136]. Worst still, up to 90% of patients in low-income countries lack access to treatment [137]. The Global Coalition for Radiotherapy has identified this huge health inequality gap and in 2022, wrote a white paper emphasising the need to draw on advances in technology including artificial intelligence, global networking, and remote solutions to help solve this underreported crisis [138]. The Global Task Force on Radiotherapy for Cancer Control (GTFRCC) quoted in 2015 that improving access to radiotherapy had the potential to save one million lives per year by 2035, along with a net macroeconomic benefit of up to US$365 billion over this 20-year period [139]. Most resource planning for LMICs looks at the benefits of radical radiotherapy in increasing cure rate and survival. The role of PRT in improving symptoms and overall quality of life is often overlooked [140]. Furthermore, many countries lack access to palliative care services and basic pain relief including morphine [141]. Elmore et al. have highlighted that integration of supportive and palliative care services alongside the improvement of radiotherapy, medical and surgical oncology infrastructure, would improve all aspects of cancer care in a holistic manner [142]. Global collaboration is needed to help to provide skilled workforce training and investment in infrastructure to help resolve this growing crisis and improve cancer care globally [143].

## 10. Radiotherapy in Specific Populations—With a Reflection of Specific Supportive Care Measures

### 10.1. Frail and Older Adults

The incidence of cancer in older populations is continuing to rise driven by increases in life expectancy globally [144]. In the UK, more than a third of new cancer cases were in people aged 75 and over, and in 2019 the incidence of cancer in those aged 75 and over made up 28.6% of new cases globally [145,146]. This is predicted to continue to rise with cancer incidence in the older population projected to reach 20.7 million globally by 2040 [147]. PRT is underutilised in the older population despite the multiple benefits including better symptom control and improved quality of life and evidence that age does not affect the likelihood of response [148,149,150,151]. This patient cohort is often underrepresented in trials despite the increasing population highlighting a growing need for research into the treatment of older adults. Barriers to PRT include multiple days of treatment, transportation and physician bias along with the challenge of multiple co-morbidities seen in this population [149]. This emphasises the need for supportive and senior adult oncology to support this patient group through treatment decisions and both SACT and radiotherapy regimes optimised to ensure the most appropriate treatment is delivered. Senior adult oncology services have been set up across some parts of the UK using an MDT approach to optimise patients prior to and during treatments, yet geographical inequalities are evident [152]. Comprehensive geriatric assessments prior to treatment have been recommended by ASCO, the RCR, and RCP, including G8 and Rockwood clinical frailty scores [153]. They help to improve treatment adherence, quality of life, and aid clinicians in complex decision-making [153,154].

### 10.2. Children and Adolescents

Children and adolescents with incurable cancer similarly face underutilisation of PRT despite its benefits and reduced intensity relative to curative treatments [127,155,156]. Factors contributing to this include a paucity of studies investigating its use, a lack of training in PRT for paediatric oncologists, the additional challenge of involving both children and parents in decision-making and logistical challenges such as travelling, time away from home and finances [127,155,156]. Furthermore, the majority of paediatric patients under the age of four require a general anaesthetic to ensure reproducible daily set-up. This generates added emotional and logistical difficulties for patients and their families and creates a risk of additional toxicities [156,157]. A response to PRT was seen in 72% of paediatric cases across a range of tumour sites in a study by Rahn et al. highlighting the significant benefit PRT can offer in this patient group [158]. Proton therapy is often used in radical paediatric treatments but Berlin et al. have looked at its use in the palliative setting in complex cases or those requiring re-irradiation [159].

### 10.3. Patients Receiving Best Supportive Care

Palliative care physicians are well placed to refer patients for PRT; however, a lack of education on this topic in their training and concerns regarding management of toxicities, along with a lack of clear pathways to a clinical oncologist for discussion are significant barriers [127,160]. Similarly, the lack of education on palliative care for clinical oncologists is felt to be a contributor [39,161]. The rise of supportive oncology as a specialty aims to overcome this with earlier involvement in the care of those with incurable cancer with the aim of prompter identification of patients who would benefit from PRT. Reinforcing this, integration of PRT with supportive care teams has been shown to improve treatment experience for patients [161]. Early supportive or palliative care involvement has been demonstrated to improve quality of life, reduce depression, and improve survival, and its incorporation has been backed by ASCO [162,163,164]. Van Oss et al. have highlighted that referral for PRT provides a good opportunity for the introduction of supportive and palliative care services if not already involved in a patient’s care [165].

The 30-day mortality in PRT has become a quality metric supported by the RCR in the UK with a recommendation that the 30-day mortality of patients receiving PRT at a centre should be less than 20% [166]. A recent meta-analysis based on international data found the 30-day mortality to be 16% and recommended this as an updated benchmark which is being recognised globally [167,168]. The aim of this recommendation is to reduce harm caused to patients from intensive multi-fraction radiotherapy treatments when their prognosis is short. However, prognostication is difficult, with one study highlighting only 40% of radiation oncologists at one American centre predicted this correctly [169]. There is a suggestion that this metric has become a barrier to some patients with a short prognosis receiving a treatment from which they could derive benefit [170]. Especially as there is evidence that a reduction in pain with PRT can be seen within 10 days and that a single fraction of radiotherapy in bone metastases has been shown to be equally as effective as multiple fractions [33,43]. Despite evidence that PRT can be beneficial near the end of life in some cases, one study found that only 3% of hospice patients received radiotherapy [171]. Integration of supportive oncology in a multidisciplinary approach within radiotherapy services could help to overcome these barriers, aiding with the difficult prognostication and decision-making in this patient cohort and helping to ensure all other symptom control options are considered before proceeding with PRT. For patients near the end of life, a simple decision-logic approach (Table 1); explicitly balancing time-to-benefit against treatment burden, favouring single-fraction or short-course PRT, and clearly defining goals of care, supports goal-concordant treatment.

**Table 1:** A table highlighting factors to consider in palliative radiotherapy decision-making in patients receiving best supportive care.

Decision	Key Questions	Practical Guidance for Decision-Making
1. Explicit Treatment Goals and Goal-Concordant Care	What is the primary symptom prompting consideration of radiotherapy (RT) (e.g., pain, bleeding, neurologic compromise, control of fungating wound)? Are goals focused on symptom relief, function preservation, or crisis management? Are these goals aligned with patient values and advance care planning?	Proceed with RT only if a clearly defined, patient-prioritised symptom is likely to be alleviated. Explicitly document how RT aligns with stated goals of care and overall supportive oncology plan. Discuss the potential conflict of symptom control vs. time with family/being at home/minimizing interventions.
2. Time-to-Benefit vs. Expected Survival	How quickly is symptom relief expected for the proposed RT indication? Is anticipated survival sufficient to realise this benefit? For example: • In bone metastases, reduction in pain seen in 10 days [43]. • In cases of bleeding, an initial response can be seen within 48 h [172]. • Median time to improvement is 1 to 2 weeks for severe impairment and 3 weeks for moderate dysfunction secondary to brain metastases [173].	Favor RT when time-to-benefit is short (days–weeks) relative to expected survival. Avoid RT when benefit is unlikely to occur within the patient’s remaining lifespan.
3. Treatment Burden and Logistics	What is the physical, logistical, and caregiver burden (transport, visits, positioning, wait times)? Is treatment feasible given performance status and care setting?	Consider RT only if treatment burden is proportionate to expected benefit. Home distance, need for medical transport, and caregiver strain should explicitly factor into decision-making.
4. Fractionation Trade-Off (Burden vs. Benefit)	Can symptom control be reasonably achieved with single-fraction RT? Does a longer course meaningfully improve outcomes in this context?	Prefer single-fraction or ultra-short-course regimens whenever clinically appropriate to minimise burden while preserving symptom relief.
5. Toxicity and Feasibility in Frail Patients	What acute toxicities may occur, and could they worsen quality of life? Are there anatomic, cognitive, or medical constraints limiting safe delivery?	Avoid RT when expected toxicity, treatment complexity, or need for immobilisation is likely to outweigh symptomatic benefit, particularly in frail or delirious patients.
6. Alternatives or Complements to RT	Are there non-radiotherapy options that may provide faster or less burdensome symptom relief (e.g., medications, procedures, supportive care interventions)?	When RT is unlikely to deliver timely benefit, prioritise alternative symptom-directed approaches and integrate palliative care consultation. RT may still be reconsidered if goals or clinical circumstances evolve.

### 10.4. Integration of Supportive Oncology Within Radiotherapy Pathways

Supportive oncology embedded within radiotherapy services provides a unifying framework that accommodates both symptom-directed and ablative paradigms while maintaining clarity of intent. In symptom-directed PRT, the primary objective is rapid symptom relief with minimal treatment burden. In contrast, oligometastatic or ablative RT, often delivered as SBRT, targets limited metastatic burden in patients with favourable disease biology and preserved performance status. Here, the aim is durable local control and, in selected cases, prolongation of progression-free and overall survival. Evidence from trials such as SABR-COMET demonstrates survival benefit at the cost of higher treatment intensity and toxicity, justifying different benefit–risk considerations [174]. Appropriate endpoints include local control, progression-free survival, overall survival, and late toxicity, rather than early symptom response alone.

Within both paradigms, supportive oncology ensures systematic symptom assessment, shared decision-making, and alignment of treatment with patient goals [36,76,100,101,102,108,109]. Explicit documentation of treatment intent at referral and throughout decision-making is critical to guide regimen selection, endpoint evaluation, and quality assessment, while optimising symptom control, toxicity mitigation, and survivorship support.

Supportive oncology integrated within radiotherapy can be operationalised through a focused set of service elements and measurable key performance indicators (KPIs) as outlined in Table 2. Prioritising outcomes that matter to patients while avoiding low-value or harmful care are key aims. This aligns with international quality frameworks, including the ASCO–ESMO consensus on quality cancer care [9], ESMO survivorship guidance [90], and MASCC–ASCO standards for advanced disease [91], which emphasise timeliness, symptom control, patient-reported outcomes, and treatment appropriateness across the cancer trajectory.

At the service level, supportive oncology can be implemented via four components: (i) **rapid multidisciplinary assessment at referral**, involving a radiation oncologist and supportive or palliative care professional [48]; (ii) **structured symptom and goals-of-care assessment** using validated patient-reported outcome measures (PROMs) [76,100,101,102]; (iii) **evidence-based treatment selection**, including hypofractionation when appropriate [29,30,36]; and (iv) **proactive post-treatment follow-up** focused on symptom response and toxicity. Unplanned hospital admissions within 14–30 days can serve as a pragmatic indicator of toxicity, unmet needs, and care coordination, consistent with evidence linking poorly controlled symptoms to increased healthcare utilisation and missed radiotherapy appointments [71,103].

Re-irradiation rates require nuanced interpretation. Higher retreatment following single-fraction radiotherapy for bone metastases is well described [33,34,35,36] but does not indicate inferior care. It reflects evidence-based strategies that reduce initial treatment burden, with retreatment offered selectively, consistent with ESTRO–EORTC guidance emphasising individualised, multidisciplinary decision-making over rigid thresholds [108,109].

Contextualised 30-day mortality and radiotherapy use in the last 30 days of life remain important quality indicators [166,167,168] but should be interpreted alongside treatment intent, fractionation, and patient-reported benefit. Structured palliative discussions during radiotherapy improve alignment with patient goals and promote appropriate end-of-life radiotherapy use [36]. Rapid symptom relief after short-course radiotherapy supports the ethical delivery of carefully selected single-fraction or ultra-hypofractionated treatments even late in the disease trajectory [43].

## 11. Conclusions

Radiotherapy remains an essential pillar of supportive oncology, offering rapid, effective symptom relief with minimal burden. Despite strong evidence, PRT is underutilised across age groups and regions, hindered by limited access, physician knowledge gaps, and inadequate multidisciplinary integration. The growing global cancer burden, rising survivorship, and technological advances intensify the need for structured supportive oncology services embedded within radiotherapy pathways. Improved prognostication, development of comprehensive toxicity management guidelines, and expansion of rapid-access PRT clinics are critical to delivering equitable, patient-centred care.

## 12. Future Directions

Key areas requiring further investigation and policy development include the following:

### 12.1. Clinical Research Needs

• Development of evidence-based guidelines for acute and late toxicity management across tumour sites.
• Prospective studies evaluating PRT outcomes in frail, geriatric, and multi-morbid patients.
• Trials defining optimal integration of SABR with supportive oncology for oligometastatic disease.
• Comparative studies on single-fraction vs. multi-fraction PRT in diverse health systems.
• Evaluation of MR-Linac in palliative settings for precision-based symptom control.

### 12.2. Supportive Oncology Program Development

• Systematic incorporation of supportive oncology teams within radiotherapy decision-making.
• Establishment of universal referral pathways between supportive and therapeutic oncology.
• Creation of dedicated radiotherapy survivorship clinics for long-term toxicity management.

### 12.3. Implementation and Global Health

• Strategies to expand radiotherapy availability in LMICs, including workforce and infrastructure development.
• Policy frameworks to support travel reimbursement programs for patients.
• Validation of predictive tools for 30-day mortality to guide ethical, patient-centred decision-making.

### 12.4. Education and Training

• Enhanced training in PRT for palliative care physicians.
• Embedded supportive oncology and palliative care training within medical and radiation (clinical) oncology curriculum.
• Multidisciplinary curricula linking supportive oncology, senior adult oncology, and radiotherapy.

## Figures and Tables

**Table 2:** A table highlighting key domains of a supportive oncology radiotherapy service with key performance indicators.

Domain	Minimal Service Element	Example KPI (Compact Set)	Rationale
Access and Timeliness	Rapid-access/one-stop-shop RT pathway (assessment, planning ± treatment same day)	Median time from referral to first fraction	Reflects responsiveness and equity of access
Decision-making	Early supportive/palliative assessment including goals-of-care discussion	Proportion with documented goals-of-care prior to RT	Anchors treatment to patient priorities
Treatment Appropriateness	Evidence-concordant fractionation	Uptake of guideline-recommended hypofractionation	Minimises burden without compromising benefit
Symptom Assessment	Routine PROM collection at baseline and follow-up	PROM completion rate (baseline and post-RT)	Addresses clinician–patient reporting discordance
Early Benefit	Structured follow-up at 7–14 days	Symptom response at 7–14 days (pain, bleeding, dyspnoea)	Captures clinically meaningful early benefit
Safety and Coordination	Proactive toxicity management and escalation pathways	Unplanned hospital admissions at 14–30 days	Surrogate for toxicity and care integration
Durability of Benefit	Contextualised assessment of retreatment	Re-irradiation rate (interpreted by prognosis/fractionation)	Reflects appropriateness, not failure
End-of-life Care	Integrated supportive oncology involvement	Contextualised 30-day mortality and RT in last 30 days of life	Guards against non-beneficial treatment while allowing appropriate short-course RT

## Data Availability

No new data were created or analyzed in this study.

## Abbreviations

The following abbreviations are used in this manuscript:

ACROP	Advisory Committee for Radiation Oncology Practice
ASCO	American Society of Clinical Oncology
ASTRO	American Society for Radiation Oncology
EORTC	European Organisation for Research and Treatment of Cancer
ESMO	European Society for Medical Oncology
ESTRO	European Society for Radiotherapy and Oncology
GTFRCC	Global Task Force on Radiotherapy for Cancer Control
LMICs	Low-and Middle-Income Countries
MASCC	Multinational Association of Supportive Care in Cancer
MDT	Multi-disciplinary team
NHS	National Health Service
OARs	Organs at Risk
PRDA	Pelvic Radiation Disease Association
PROMs	Patient Reported Outcome Measures
PRT	Palliative radiotherapy
RCR	Royal College of Radiologists
RCP	Royal College of Physicians
RT	Radiotherapy
SABR	Stereotactic ablative body radiotherapy
SACT	Systemic anticancer therapy
SBRT	Stereotactic body radiation therapy
SFRT	Spatially fractionated radiotherapy
WBRT	Whole-brain radiotherapy
WHO	World Health Organisation
UK	United Kingdom
